# Bridging structural MRI with cognitive function for individual level classification of early psychosis *via* deep learning

**DOI:** 10.3389/fpsyt.2022.1075564

**Published:** 2023-01-10

**Authors:** Yang Wen, Chuan Zhou, Leiting Chen, Yu Deng, Martine Cleusix, Raoul Jenni, Philippe Conus, Kim Q. Do, Lijing Xin

**Affiliations:** ^1^Key Laboratory of Digital Media Technology of Sichuan Province, School of Computer Science and Engineering, University of Electronic Science and Technology of China, Chengdu, Sichuan, China; ^2^Animal Imaging and Technology Core, Center for Biomedical Imaging, Ecole Polytechnique Fédérale de Lausanne, Lausanne, Switzerland; ^3^Laboratory for Functional and Metabolic Imaging, Ecole Polytechnique Fédérale de Lausanne, Lausanne, Switzerland; ^4^Institute of Electronic and Information Engineering of UESTC in Guangdong, Dongguan, Guangdong, China; ^5^Department of Biomedical Engineering, King's College London, London, United Kingdom; ^6^Department of Psychiatry, Center for Psychiatric Neuroscience, Centre Hospitalier Universitaire Vaudois and University of Lausanne, Lausanne, Switzerland; ^7^Service of General Psychiatry, Department of Psychiatry, Centre Hospitalier Universitaire Vaudois and University of Lausanne, Lausanne, Switzerland

**Keywords:** early psychosis, cognition estimation, classification, deep learning, structural MRI (sMRI), schizophrenia, cognition function

## Abstract

**Introduction:**

Recent efforts have been made to apply machine learning and deep learning approaches to the automated classification of schizophrenia using structural magnetic resonance imaging (sMRI) at the individual level. However, these approaches are less accurate on early psychosis (EP) since there are mild structural brain changes at early stage. As cognitive impairments is one main feature in psychosis, in this study we apply a multi-task deep learning framework using sMRI with inclusion of cognitive assessment to facilitate the classification of patients with EP from healthy individuals.

**Method:**

Unlike previous studies, we used sMRI as the direct input to perform EP classifications and cognitive estimations. The proposed deep learning model does not require time-consuming volumetric or surface based analysis and can provide additionally cognition predictions. Experiments were conducted on an in-house data set with 77 subjects and a public ABCD HCP-EP data set with 164 subjects.

**Results:**

We achieved 74.9 ± 4.3% five-fold cross-validated accuracy and an area under the curve of 71.1 ± 4.1% on EP classification with the inclusion of cognitive estimations.

**Discussion:**

We reveal the feasibility of automated cognitive estimation using sMRI by deep learning models, and also demonstrate the implicit adoption of cognitive measures as additional information to facilitate EP classifications from healthy controls.

## 1. Introduction

Artificial intelligence (AI) approaches, particularly machine learning (ML) and deep learning (DL), have been extensively studied to accelerate medical data analysis and assist clinical interventions in many pathological contexts (1, 2). Many applications have been conducted in psychiatric disorders using neuroimaging measures [e.g., sMRI (3)] as input and incorporated with AI models (e.g., supported vector machine and artificial neural networks) to establish automated diagnostic workflows at a single subject level (4, 5). Previous machine learning works in schizophrenia have used handcrafted features extracted from sMRI data to distinguish patients from healthy individuals (6), but such feature extraction process usually involves a long computational time. To reduce computational cost, recent efforts have focused on using directly sMRI images as input, and promising results have been achieved with the help of the latest AI models (e.g., convolutional neural networks, CNNs) (7, 8). However, these studies have mainly focused on patients at chronic stage, the classification of early psychosis (EP) patients from healthy controls (HCs), is considered to be more challenging (9–12), because the brain structural changes in patients with EP are mild and not evident, making computer-aided classification methods less robust and accurate.

Furthermore, progressive cognitive deficit is one major feature of schizophrenia (13–15), inspiring the possibility of using individual cognition levels, in addition to sMRI images, to facilitate automated classification of patients with EP from HCs. Several recent studies have used the DL framework to incorporate cognitive estimation into the workflow to facilitate the diagnosis of Alzheimer's disease by explicitly including cognitive measures as secondary inputs (16, 17). However, this approach requires additional cognitive assessment that is not part of routine neuropsychiatric clinical examinations. Moreover, although several studies have been done using sMRI images to identify individual cognitive impairments *via* DL (18, 19), to the best of our knowledge, no study has been done to incorporate cognition estimation for classifying patients with EP and controls.

**Figure 1:**
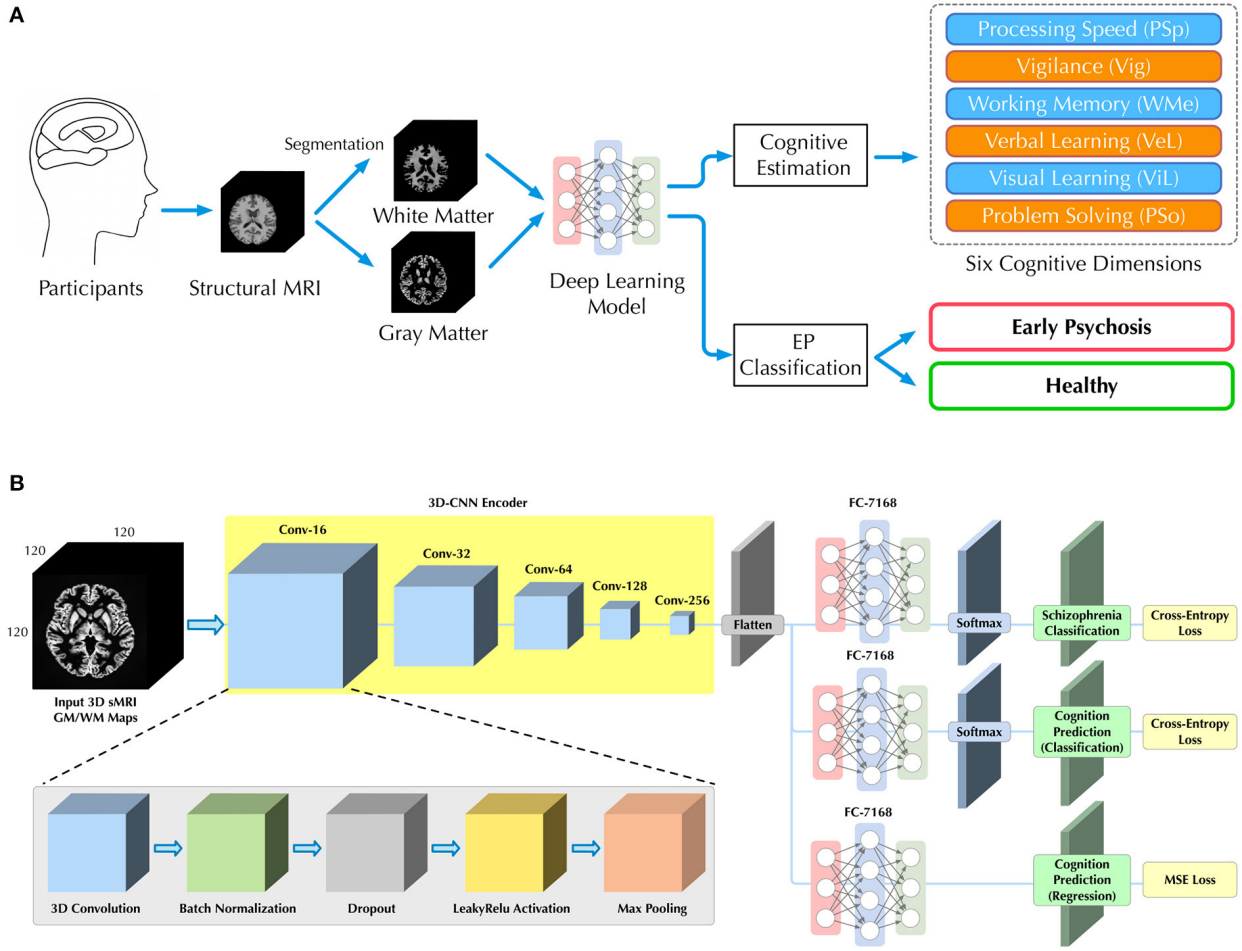
Illustrations of **(A)** our workflow for classification of patients with EP from HCs and cognitive estimation on six dimensions, and **(B)** the deep learning architecture with a 3D-CNN feature encoder and three independent MLP subbranches for different subtasks of EP classification and cognitive estimations.

Therefore, in this study, we aim to apply a multi-task DL model by using sMRI as an input to classify patients with EP from healthy controls and to simultaneously predict cognition levels at the single subject level. We further investigated whether the inclusion of cognitive levels estimation could facilitate the classification for patients with EP and controls. Specifically, as shown in Figure 1B, a three-dimensional convolutional neural network (3D-CNN) is used to learn discriminative structural features directly from sMRI arrays. Then, three multilayer perceptron (MLP) subbranches are used to perform EP/HC classifications and cognition estimations. We evaluate the proposed model on an in-house data set, consisting of 77 sMRI 3D arrays (38 patients with EP, 39 HCs).

## 2. Materials and methods

### 2.1. Problem setup

As shown in Figure 1A, given the sMRI image, we seek to estimate participant's cognitive level and classify patients with EP from healthy individuals in a fully automated manner. Unlike previous studies (16, 20–22), we directly utilized sMRI images as input without additional imaging analysis (e.g., voxel-based morphometry), which allowed us to more natively understand how brain structure itself contributes to the EP classification and cognition estimation.

### 2.2. Materials and data set

#### 2.2.1. Participants

sMRI data and corresponding neurocognitive scores were obtained from Department of Psychiatry at the Lausanne University Hospital (CHUV). The data set consists of 38 patients with EP and 39 healthy controls (HC). Detailed demographic information of all participants are shown in Table 1. Specifically, the Positive and Negative Syndrome Scale (PANSS) was provided as the sum of positive, negative and general PANSS values. The patients with EP were recruited from the TIPP Program (Treatment and Early Intervention in Psychosis Program, University Hospital, Lausanne, Switzerland) (23). All the participants provided informed written consent for this study, and the procedure was approved by the local Ethics Committee (Commission cantonale déthique de la recherché sur lêtre humain - CER-VD), in accordance with the Declaration of Helsinki. Detailed recruitment criteria for participants can be found in Supplementary material A.

**Table 1:** Demographic information and neurocognition performance of 77 subjects.

**Variable**	**Total (*n* = 77)**	**HC (*n* = 39)**	**EP (*n* = 38)**	***P*** **-value**
Age,mean (SD), y, *t*	24.42 (4.81)	24.54 (5.35)	24.29 (4.23)	0.884
Max	43.69	43.69	40.57	-
Min	16.22	16.22	18.30	-
Gender, χ^2^				0.817
Male	62	31	31	-
Female	15	8	7	-
Ethnicity, χ^2^				0.852
Caucasian	56	28	28	-
Other	21	11	10	-
Duration of psychosis, mean (SD), y	-	-	1.00 (0.73)	-
Clinical Scores				
Positive PANSS, mean (SD)	-	-	11.61 (3.45)	-
Negative PANSS, mean (SD)	-	-	16.04 (5.66)	-
**Cognitive Scores**				
Processing speed (PSp)^‡^, mean(SD), *t*	46.72 (12.97)	55.00 (9.54)	38.23 (10.51)	1.75E-05
Vigilance (Vig), mean (SD), *t*	47.07 (10.46)	51.16 (5.50)	42.88 (12.70)	0.0651
Working memory (WMe), mean (SD), *t*	49.67 (8.29)	50.00 (6.79)	49.35 (9.77)	0.556
Verbal learning (VeL)^‡^, mean (SD), *t*	49.23 (8.43)	53.81 (6.91)	44.54 (7.37)	4.12E-04
Visual learning (ViL)^‡^, mean (SD), *t*	47.83 (9.05)	51.31 (7.54)	44.27 (9.30)	0.111
Problem solving (Pso), mean (SD), *t*	53.33 (8.12)	57.15 (4.57)	49.42 (9.22)	0.0801

‡ with significant difference between groups (*p* < 0.05);

SD, standard deviation; PANSS, Positive and Negative Syndrome Scale; χ^2^, Chi-squared test; *t*, two-tailed student *t*-test; y, year.

#### 2.2.2. Structural MRI acquisition

Patients and controls underwent magnetic resonance imaging at a 7 Tesla/68 cm MR scanner (Siemens Medical Solutions, Erlangen, Germany). A 32-channel receive coil (NOVA Medical Inc., MA) with a single channel volume transmit coil was used. 3D T1-weighted MR images were acquired using MP2RAGE (TE/TR = 1.87/5,500 ms, TI1/TI2 = 750/2,350 ms, α1/α2 = 4°/5°, slice thickness = 1 mm, FOV = 240 × 256 × 160 mm^3^, voxel size = 1 mm^3^ isotropic, bandwidth = 240 Hz/Px) (24). The original dimension of acquired sMRI data array is 240 × 256 × 160.

#### 2.2.3. Preprocessing

To generate appropriate inputs, we performed preprocessing of sMRI data using CAT12 toolkit for estimation of the probability maps of white matter (WM) and gray matter (GM). Skull striping and registration to standard space with MNI152 template were performed. Then, probability maps of WM and GM were generated after tissue segmentation and bias correction. The resulting WM and GM probability maps were down-sampled to 120 × 120 × 120 for computational efficiency.

#### 2.2.4. Neurocognitive measures

The MATRICS Consensus Cognitive Battery (MCCB) (25, 26) was assessed for both EP and HC groups, excluding the Mayer-Salovey-Caruso Emotional Intelligence Test (MSCEIT), which does not “translate” well into French as an index of social cognition. The neurocognitive measures include six dimensions, i.e., processing speed (PSp), vigilance (Vig), working memory (WMe), verbal learning (VeL), visual learning (ViL) and problem solving (PSo). There exists some missing entries in the cognitive assessment data, so we replaced all missing data with the average values to generate proper training data (17). The quantity of missing entries is: PSp 5, Vig 3, WMe 4, VeL 1, ViL 1, and PSo 1. There is at most one missing cognitive dimension per subject. The distribution of scores for all cognitive dimensions are shown in Table 1. Two-tailed student *t*-test was performed between the two groups, and significant difference was found on PSp, VeL and ViL with a *p* < 0.05.

As pointed out by previous studies (19, 27), the estimation of cognitive level can be done by either classification or regression, that classification task is to manually classify continuous scores into different discrete categories and predict the probability of which category each case should be in, whereas regression is a direct prediction of scores. In this study, for the classification task, we evenly divided the scores between the maximum and minimum values into *n* equal parts, *i.g*., *n* categories. It is worth noting that since the maximum and minimum values are different for each cognitive assessment, the interval is also different among the *n* categories. Normally, larger *n* represents a more fine separation of cognitive levels and greater difficulty in prediction.

#### 2.2.5. External data set

Besides the in-house data set, we also performed experiment on a second data set, which is from the project of HCP-Early Psychosis (HCP-EP) Release 1.1 from Human Connectome Projects^1^ and the Adolescent Brain Cognitive Development^SM^ (ABCD) Study, held in the NIMH Data Archive (NDA). Detailed recruiting criteria can be found in this website.^2^ Detailed cognition measuring methods can be found in this website.^3^ Only sMRI data was used as input. For the estimation of cognition, we chose six dimensions of cognitive measures that were closest to our in-house dataset, which are the age adjusted scores from NIH Toolbox Dimensional Change Card Sort Test, NIH Toolbox Flanker Inhibitory Control and Attention Test, NIH Toolbox List Sorting Working Memory Test, Pattern Comparison Processing Speed Test, Seidman Auditory CPT test and NIH Toolbox Picture Vocabulary Test. After filtering, a total of 164 subjects had both sMRI data and cognitive scores.

### 2.3. Proposed method

#### 2.3.1. 3D-CNN multi-task learning framework

In this study, 3D sMRI arrays were directly used as input for classifications, so we applied 3D-CNN models as a deep learning architecture to encode visual features, similar in previous studies (8, 28, 29). Instead of dividing the sMRI array into 2D images and using 2D-CNN (18, 30) for feature encoding, 3D-CNN can consider all inputs at once to better capture local features in the 3D space and contribute to the final classification.

To predict both the cognitive level and the probability of EP for each participant, we further introduced a multi-task learning framework. Based on the same visual features extracted by the 3D-CNN, three independent MLP networks were used as individual subbranches for different tasks, including EP classification, cognitive level classification (CLC) and cognitive level regression (CLR). The complete architecture of our 3D-CNN encoder and multi-task learning framework is depicted in Figure 1B and corresponding details are provided in Table S7 in Supplementary material D. The sequential structure of our 3D-CNN encoder was inspired by the previous study on schizophrenia classification (8).

#### 2.3.2. Multi-channel 3D array input

We consider the GM and WM probability maps as two different feature channels and make channel concatenations to generate a single 3D array as the input to our model. Unlike previous study (8), where different segmentation components were used as multiple inputs and fed into a model in parallel, our multi-channel 3D array helps to reduce the training parameters and retain all the information from GM and WM. In this case, the dimension of input 3D array will be *H* × *W* × *D* × 2, where *H*, *W*, *D* denotes height, width, depth and 2 is the number of channels. The full volume of size 120 × 120 × 120 × 2, rather than smaller volume patches, was used for training and testing. Furthermore, in experiments where only GM or WM is used for training, a single probability map will be replicated once to remain the dimensionality of the input 3D array.

#### 2.3.3. End-to-end training

Our framework is an end-to-end deep learning system and thus several loss functions were used to train the proposed model for parameter updating. Specifically, for classification tasks (i.e., EP and cognitive level classification), the conventional cross entropy (CE) loss is used, which is defined as


(1) $$\begin{array}{l} L_{CE}=-\overset{c}{\underset{i=1}{\sum }}s^{i}\log\left({ŝ}^{i}\right), \end{array}$$

where *s* is the true label, ŝ is the prediction, and *c* is the number of class. For the task of cognition regression, the mean square error (MSE) loss is used, which is defined as


(2) $$\begin{array}{l} L_{MSE}=||g-ĝ|{|}_{2}^{2}, \end{array}$$

where *g* and ĝ denote ground truth label and prediction, respectively. The final loss function is defined as:


(3) $$\begin{array}{l} L_{loss}=L_{CE-SZ}+L_{CE-C}+L_{MSE}+L_{reg}, \end{array}$$

where $L_{CE-SZ}$ denotes CE loss for EP classification, $L_{CE-C}$ denotes CE loss for cognitive level classification, $L_{MSE}$ denotes MSE loss for cognitive level regression, and $L_{reg}$ represents the regularization loss [or weight decay (31)] used to avoid overfitting. As an end-to-end framework, training losses are back-propagated from three multi-task subbranches to the 3D-CNN, updating the parameters of the entire network with an optimization algorithm [e.g., Adam (32)]. Finally, through minimizing the $L_{loss}$, the network could learn a nonlinear mapping from the input 3D sMRI array to EP and cognitive state, enabling EP classification and cognitive estimation for unseen individuals.

#### 2.3.4. Gender influence

Since gender differences were found to be important in WM and GM of psychosis (33–35), and due to the uneven gender distribution of the in-house dataset, two experiments were designed to assess how gender difference affects the performance of the DL-based model on cognitive estimation and EP classification. First, gender information was encoded as an orthogonal embedding and explicitly fed into the model along with the sMRI scan. Second, subjects were divided into two gender subgroups, and experiments were conducted separately for each subgroup.

### 2.4. Competing methods

#### 2.4.1. Deep learning-based model

Apart from 3D-CNN, we also used a 2D-CNN framework, similar to the model of Jiang et al. (18) and Li et al. (5), for comparison. The latest lightweight 2D convolutional architectures, MNasNet (36), and a cumbersome model, ResNet-18 (37), were used as the feature encoders since they have been commonly used in previous studies (3, 5, 8, 38). In a 2D-CNN framework, for each participant, image features are extracted slice by slice and concatenated for final classification, which introduces more computational cost than the 3D-CNN model. Furthermore, since 3D-CNNs do not have pre-trained weights like 2D-CNNs, all 3D-CNNs models were trained from scratch. Nevertheless, results are reported for 2D-CNNs with and without pre-trained weights.^4^

#### 2.4.2. Handcrafted feature-based machine learning

To compare with the proposed DL workflow, we also performed the classification tasks with several latest ML methods. The GM and WM probability maps were flattened into feature vectors and the principal component analysis (PCA) was used for dimensionality reduction to produce proper training inputs for ML models. Besides the WM and GM maps, volumetric and surface analysis was also performed with CAT12 toolkit to calculate region of interest (ROI) volumes and cortical surface thickness as handcrafted features for comparison. We adopted the analysis with default settings and obtained 388 ROI volume features and 219 cortical thickness features after filtering out the null values. The Cobra^5^ and neuromorphometircs^6^ were used as ROI atlas. Dimensionality reduction was also performed on handcrafted features to make them the same size as GM/WM-based features. We selected several popular ML models for comparison, including random forest (RF), supported vector machine (SVM) and gradient boost machine (GBM).

### 2.5. Implementation details

All models were implemented with the Python (version 3.7) programming language and several free Python-based packages. For ML models, the GBM was implemented with a popular lightGBM^7^ framework and other models were implemented using scikit-learn toolkit (39). The number of estimators in RF model was set as 500 and radial basis function kernel was used in SVM model.

We used PyTorch (version 1.6 stable) as the DL framework to implement all DL-based models. The Adam (32) was used as the optimizer with a starting learning rate of 1e-4, and the learning rate was made to decay by 0.7 after every 60 epochs to help reach optima. Data augmentation (random rotation and flipping) and weight decay of the optimizer (at a rate of 0.02) were used as data set expansion and regularization, respectively, to help prevent overfitting. The batch size was set to 10, and 300 epochs were used. All experiments were conducted on an Ubuntu 18.04 system with two NVIDIA GeForce RTX 2080 Ti graphical processing unit (GPU) and 22 gigabytes memory. The versions of Compute Unified Device Architecture (CUDA) and the driver for the GPU were 10.2 and 460.73.01, respectively. We used a grid search strategy to determine the hyperparameters with learning rates in the range of [1e-3, 1e-4, 1e-5], batch sizes in the range of [4, 8, 10, 12], and weight decay in the range of [0.0, 0.1, 0.2, 0.3, 0.4].

### 2.6. Evaluation metrics

We used accuracy, *F*_1_-score, specificity and area under curve (AUC) of receiver operating characteristic (ROC) as the metrics to evaluate the classification performance. Specifically, the *F*_1_-score is the harmonic mean between recall (sensitivity) and precision. The accuracy, *F*_1_-score and specificity are respectively defined as $Accuracy\left(acc\right)=\frac{tp+tn}{tp+fn+fp+tn},$ $F_{1}\text{-}score\left(F_{1}\right)=\frac{2\times tp}{2\times tp+fp+fn}$ and $Specificity\left(spe\right)=\frac{tn}{fp+tn},$ where *tp*, *fp*, *tn* and *fn* refer to true positive, false positive, true negative, and false negative, respectively. While *F*_1_-score mainly focus on evaluating prediction performance on positive targets (i.e., the EP cases), the specificity focus on evaluating the negative ones (i.e., the healthy cases). All these metrics range from 0 to 1, with higher metrics indicating better predictive performance achieved by the model. In addition, we adopted mean absolute error (MAE) and coefficient of determination (R^2^) as metrics to evaluate regression performance, which is defined as $MAE=\frac{1}{m}\overset{m}{\underset{i=1}{\sum }}|y_{i}-{\hat{y}}_{i}|$ and $R^{2}=1-\frac{\overset{m-1}{\underset{i=0}{\sum }}{\left(y_{i}-{\hat{y}}_{i}\right)}^{2}}{\overset{m-1}{\underset{i=0}{\sum }}{\left(y_{i}-{ȳ}_{i}\right)}^{2}},$ where *m* denotes number of samples, *y* and $\hat{y}$ denote ground truth and prediction, respectively.

### 2.7. Reduce evaluation bias *via* cross validation

Since the size of the data set is relatively small for a deep learning model, we applied a five-fold cross-validation strategy in this study in order to thoroughly evaluate and avoid overfitting. There were 77 3D sMRI arrays after pre-processing. These samples were divided into five parts equally, and one part of them was selected one by one as the test set and the rest as the training set. Stratified sampling was used to ensure that the gender ratio in the training/test groups was the same. After that, all metrics are presented as the mean and standard deviation of the five experiments. In this work, the multi-task deep learning framework accomplished two tasks including cognitive estimation and EP classification.

## 3. Results

### 3.1. Results for cognition estimation and EP classification

#### 3.1.1. Cognition estimation

**Figure 2:**
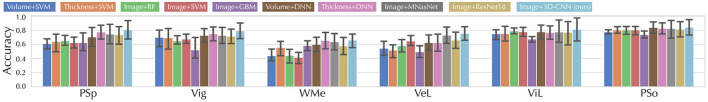
Accuracy of our model in two-categorized CLC task compared with different ML and DL counterparts in six cognitive estimation dimensions. All the DL models shown were trained from scratch.

We first evaluate the cognitive estimation performance of the proposed method and competing methods in terms of CLC task, of which results are shown in Table S1 in Supplementary material B. Specifically, the 3D-CNN model achieved *F*_1_-scores of 70.1 ± 3.5%, 51.9 ± 8.1%, 31.9 ± 7.5%, 16.2 ± 3.7% in the two-, three-, five-, and 10-categorized CLC tasks, respectively. Furthermore, we present the classification accuracy for each cognition estimation dimension while *n* = 2 in Figure 2 and the regression results for cognitive estimation (i.e., CLR) in Table S1 in Supplementary material B. The 3D-CNN model achieved a R^2^ of –0.878 ± 0.121 and a MAE of 8.567 ± 1.950, while the *Volume + SVM* combination achieved the best CLR results with a R^2^ of -0.086 ± 0.139 and a MAE of 7.299 ± 1.735. We also presented results on an external data set (Table S4 in Supplementary material B), discussed the effects of using Huber loss on the CLR in Tables S2, S5 in Supplementary material B, and discussed the effects of gender differences on the cognition estimation (Table S8 in Supplementary material D).

#### 3.1.2. EP classification

**Figure 3:**
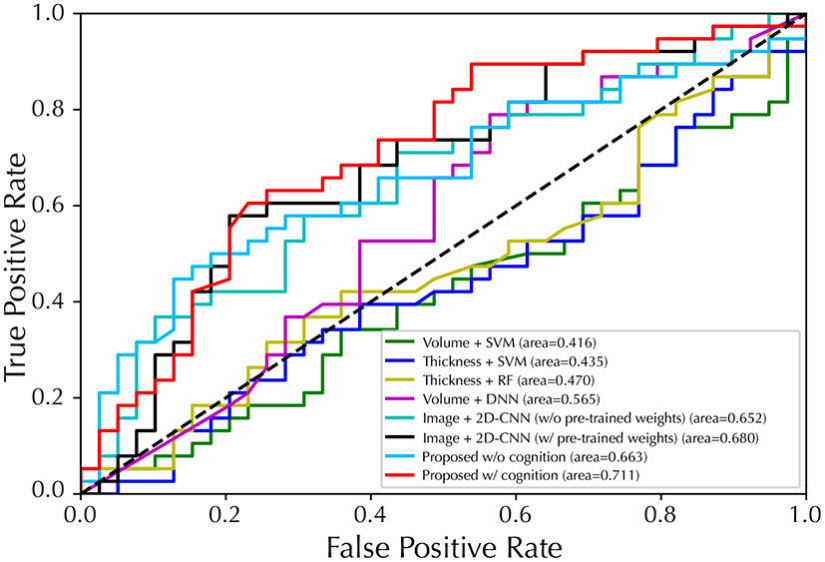
Performance of ROC curves for EP/HC classification with five-fold cross-validation. The proposed model used GM map as input.

For the second task of EP classification, our model was compared with several latest counterparts (5, 40–43), of which models were re-implemented based on the settings of the original publications. The results of EP classification are shown in Figure 3 and Table 2. Specifically, the *Thickness features + SVM* combination achieved the best results in ML methods with an accuracy of 58.4 ± 9.0%, a *F*_1_-score of 60.8 ± 10.6%, and a specificity of 60.4 ± 10.1%, while the proposed method achieved an accuracy of 74.9 ± 4.3%, a *F*_1_-score of 74.5 ± 4.2%, and a specificity of 82.3 ± 6.3% with the inclusion of cognitive estimation. We also presented results on an external data set (Table S4 in Supplementary material B), discussed the computational costs of models in Table S6 in Supplementary material C, and discussed the effects of gender differences on the EP classification (Table S9 in Supplementary material D).

**Table 2:** Comparison on sMRI-based studies for EP classification.

**Method**	**acc**	**F_1_**	**spe**
Volume features + SVM (40)	50.8 ± 5.6	54.1 ± 8.8	53.4 ± 7.3
Volume features + DNN (41)	65.7 ± 13.4	69.0 ± 11.8	69.5 ± 13.3
Thickness features + SVM (42)	58.4 ± 9.0	60.8 ± 10.6	60.4 ± 10.1
Thickness features + RF (43)	50.7 ± 13.5	55.5 ± 11.7	54.6 ± 10.7
sMRI images + 2D-CNN (5)	68.9 ± 5.6	69.4 ± 5.1	71.0 ± 5.7
Proposed w/o cognitive estimation^†^	70.6 ± 4.1	70.5 ± 4.1	75.3 ± 5.8
Proposed w/ cognitive estimation^†^	73.5 ± 3.3	74.2 ± 3.0	80.1 ± 5.1
Proposed w/o cognitive estimation^‡^	71.0 ± 4.3	70.1 ± 4.4	73.8 ± 5.9
Proposed w/ cognitive estimation^‡^	**74.9 ±4.3**	**74.5 ±4.2**	**82.3 ±6.3**

All results are shown in percentage and the best results are highlighted in bold.

† WM and GM inputs;

‡ GM input.

As we hypothesized that the introduction of a cognitive classification task could bring features about individual brain structure to the DL model, it remains unclear whether more classification categories could lead to more discriminative features for EP classification. Therefore, we divided cognitive scores into different number of categories in the CLC subtask and assessed how this would affect classification performance for EP, the results are shown in Figure S1 in Supplementary material B.

#### 3.1.3. Validation on ABCD HCP-EP data set

The experiments of cognition estimation and EP classification were also performed on the external ABCD HCP-EP data set. For cognition estimations (Table S3 in Supplementary material B), the proposed method obtained *F*_1_-scores of 81.6 ± 1.8%, 61.4 ± 5.9%, 40.3 ± 6.8% on the two-, three-, and five-categorized CLC tasks, respectively. A *R*^2^ of 0.074 ± 0.499% was achieved by the proposed method on the CLR task. For EP classification (Table S4 in Supplementary material B), the proposed method achieved an accuracy of 75.9 ± 5.3% and an *F*_1_-score of 84.1 ± 5.2% when using WM and GM as inputs, and achieved an accuracy of 75.8 ± 6.1% and an *F*_1_-score of 84.3 ± 5.1% when using only GM as input. After the inclusion of cognition estimation, the accuracy was improved by 2.2% and the *F*_1_-score was improved by 3.1% when using WM and GM as inputs, and the accuracy was improved by 2.9% and the *F*_1_-score was improved by 2.8% when using only GM as input.

#### 3.1.4. Gender influence study

Then, the influence of gender difference was elaborated *via* two experiments. For the first experiment where gender information was fed into the model along with sMRI scan (Table S8 in Supplementary material D), the 3D-CNN model achieved a *R*^2^ of –0.885 ± 0.126 and a *F*_1_-score of 70.0 ± 3.5 on cognition estimation task after adding the gender embeddings. The metric difference is 0.007 (*R*^2^) and 0.1 (*F*_1_-score) compared to the 3D-CNN model without the gender information. For other DL-based methods, the performance differences are: for *Image + MNasNet*, 0.002 (*R*^2^) and 0.1 (*F*_1_-score); for *Image + ResNet-18*, 0.004 (*R*^2^) and 0.1 (*F*_1_-score). Besides, the proposed method with cognition estimation and gender information achieved an accuracy of 74.8 ± 4.3 and a *F*_1_-score of 74.5 ± 4.3 on EP classification task. The metric difference is 0.1 (acc) and 0.0 (*F*_1_-score) compared to the one without the gender information. For the second experiment, subjects were divided into two subgroups based on gender and results of EP classification task were presented (Table S9 in Supplementary material D). In the male subgroup, the proposed method using GM input achieved an accuracy of 74.6 ± 6.7 and a *F*_1_-score of 74.1 ± 6.0 with the inclusion of cognition estimation subtask, whereas accuracy decreased to 70.8 ± 6.4 and *F*_1_-score decreased to 70.0 ± 5.6 without the inclusion. In the female subgroup, the proposed method using GM input achieved an accuracy of 68.7 ± 10.2 and a *F*_1_-score of 69.0 ± 8.9 with the inclusion of cognition estimation subtask, whereas accuracy decreased to 66.4 ± 9.9 and *F*_1_-score decreased to 66.1 ± 10.4 without the inclusion.

#### 3.1.5. Ablation study

Furthermore, ablation studies on WM/GM inputs and CLC/CLR subtasks were conducted. First, we evaluated the effect of using different sMRI images (i.e., WM or GM images) as input on the EP classification to evaluate how they contribute to the classification in the context of DL, the results are shown in Table 3. With both WM and GM as inputs, the proposed method achieved *F*_1_-scores of 70.5 ± 4.1%, 72.5 ± 4.0%, 73.5 ± 4.0%, and 74.2 ± 3.0% for EP classification, EP classification with CLC subtask, EP classification with CLR subtask, and EP classification with CLC and CLR subtasks, respectively. The best *F*_1_-score result of 74.5 ± 4.2% was obtained when using GM as input with the CLR as the subtask. We then evaluated how different ways of introducing the cognitive assessment subtask (i.e., CLC or CLR) contributed to the classification of EP, the results are shown in Table 4. Specifically, our model with the CLR subtask achieved the best EP classification results with an accuracy of 74.9 ± 4.3%, *F*_1_-scores of 74.5 ± 4.2%, specificity of 82.3 ± 6.3%, and AUC of 71.1 ± 4.1%. With only the CLC subtask, the results decreased with an accuracy of 71.4 ± 3.7%, *F*_1_-scores of 70.2 ± 5.4%, specificity of 74.4 ± 5.4%, and AUC of 67.4 ± 4.5%.

**Table 3:** Results of *F*_1_-score (%) for EP classification.

**Input**	**Task**			
	**EP**	**EP + CLC**	**EP + CLR**	**EP + CLC + CLR**
WM	63.2 ± 4.1	64.6 ± 4.3	64.2 ± 4.4	63.5 ± 4.6
GM	70.1 ± 4.4	70.2 ± 5.4	**74.5 ±4.2**	71.2 ± 3.8
WM + GM	**70.5 ±4.1**	**72.5 ±4.0**	73.5 ± 4.0	**74.2 ±3.0**

Here EP denote early psychosis classification. The *n* was set to five for CLC. The best results are in bold.

**Table 4:** EP classification performance of our model when introducing different cognitive estimation subtasks, using GM images as input.

**Model with Task**			**acc**	**F_1_**	**spe**	**AUC**
**EP**	**CLC**	**CLR**				
✓			71.0 ± 4.3	70.1 ± 4.4	73.8 ± 5.9	66.3 ± 3.9
✓	✓		71.4 ± 3.7	70.2 ± 5.4	74.4 ± 5.4	67.4 ± 4.5
✓		✓	**74.9 ±4.3**	**74.5 ±4.2**	**82.3 ±6.3**	**71.1 ±4.1**
✓	✓	✓	71.1 ± 3.9	71.2 ± 3.8	77.0 ± 5.9	68.0 ± 4.9

Here EP denotes early psychosis classification. The *n* was set to five for CLC. The bold values indicate the best results of each metric under different task settings.

#### 3.1.6. Qualitative illustration

**Figure 4:**
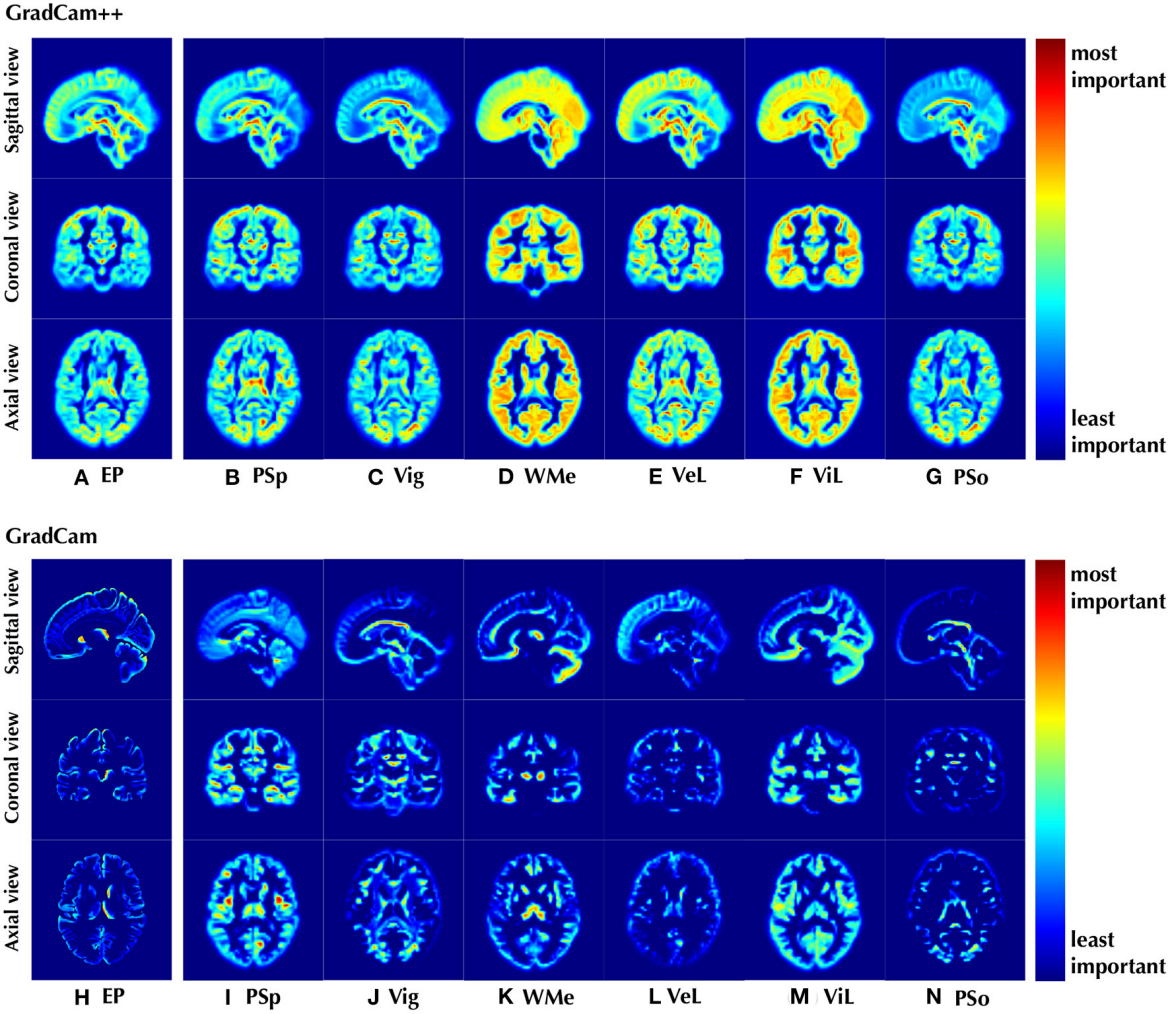
Visualization of the discriminative positions identified by the proposed model on **(A, H)** EP classification and **(B–G, I–N)** CLC tasks with attentional weights. The results were shown as the mean of all cases in the data set. We used both WM and GM images as input and *n* was set to two for CLC.

Last but not least, our proposed framework could potentially identify brain regions that may be associated with psychosis, thus we present the attention maps using GradCam++ (44) and GradCam algorithms (45, 46) in Figure 4 to illustrate brain structures of importance. Besides structural biomarkers for psychosis, we also demonstrate the attention maps for CLC in Figures 4B–G, I–N.

## 4. Discussion

Despite the recognized brain structural alterations (47–49) and cognitive deficits (13–15) in schizophrenia, no studies have performed sMRI-based cognitive estimation in EP, nor have cognitive measures been incorporated into EP classification. In the present work, a multi-task deep learning framework using sMRI was used to bridge sMRI and cognitive estimation for improving the classification performance of EP, which can automatically capture structural features from 3D sMRI scans for EP classification and provide cognition as supporting evidence at individual level within a unified framework. While most of ML-based classifiers relied on features of time-consuming volumetric or surface based analysis, the proposed method performs EP/HC classifications and cognition estimation using only sMRI as input. By comparison with the latest models and ablation studies, we revealed the feasibility of automatic cognitive estimation at the individual level and demonstrated the implicit adoption of cognitive measures as additional information could facilitate EP classification from healthy controls. Furthermore, the main structural contributors involved in the process of EP classification and cognitive estimation are identified.

### 4.1. Cognitive estimation performance

For CLC task, our model achieves better CLC performance in most cases. For example, our model obtains the best *F*_1_-score of 70.1% on the two-categorized (i.e., *n* = 2) CLC task. Same results can be observed on the three- and ten-categorized (*n* = 3 and *n* = 10) CLC that our method outperforms all other counterparts with significant margins. Although in the only case (*n* = 5) our method did not get the first place, we still got the second best performance. Based on these results, it can be seen that the proposed DL model was able to classify individuals' cognitive states into groups using sMRI and achieved promising performance on two-categorized CLC task with higher accuracy than chance.

Specifically for the two-categorized CLC task, all DL-based models achieved better performance than ML-based models in all cognitive dimensions. Although the DL-based models using sMRI images as input performed similarly across the four cognitive dimensions (PSp, Vig, ViL, and PSo), it is noteworthy that our method achieved significant improvements in the WMe and VeL dimensions. Thus, our method performs most convincingly for CLC task in all six dimensions and even achieves an accuracy of more than 80% in some dimensions (PSp, ViL, and PSo). Since our method uses sMRI images directly as input without further volumetric and cortical surface based analysis, it achieves both the overall best classification performance and efficiency, both of which are crucial for clinical translations.

However, the CLR performance of all models was worse than expected, even worse than random guesses (*R*^2^ ≤ 0.0). A possible reason for the poor regression performance may be due to the limited sample size of the data set (50). By comparing the performance of DL and ML models, it can be seen that DL models generally performed worse than ML models. This suggests that DL models may be more sensitive to the lack of samples (19), and that DL models may be more suitable for classification rather than regression in a sample-limited context.

Furthermore, a considerable performance improvement is observed by comparing the results of the in-house dataset with the external ABCD HCP-EP dataset (Table S3 in Supplementary material B). First, for the two-categorized CLC task, our method achieved an *F*_1_-score of 81.6 ± 1.8 on the external dataset, which is an 11.5% improvement compared to the *F*_1_-score on the in-house dataset (70.1 ± 3.5). Second, for the CLR task, most DL-based methods achieved better than random guesses (*R*^2^ > 0.0) results and our method achieved the best one (0.074 ± 0.499), which improved by 0.952 compared to the *R*^2^ on in-house dataset (–0.878 ± 0.121). The improved performance is most likely due to the fact that the external dataset (*n* = 164) has more than twice the amount of data as the in-house dataset (*n* = 77), and more data allows the model to better grasp structural information from the input and learn correlations between sMRI scans and cognitive levels. Given that the data from the external dataset were obtained in a different imaging pipeline and were composed of subjects from cohorts with different age and gender distributions, the results again demonstrate the validity of individual-level cognitive estimation using DL-based models, especially when more data are available.

### 4.2. Early psychosis classification performance

For EP classification, our proposed model generally outperforms the other five competing methods in all metrics. For instance, our model using solely sMRI images (with GM as input) achieved the best F_1_-score (74.5%) compared to ML-based models using volumetric features [54.1% (40)] and cortical thickness features [55.5% (42) and 60.8% (43)]. In addition, our 3D-CNN model also achieves better performance in all metrics compared to 2D-CNN (5), indicating that features are extracted directly from 3D sMRI arrays more efficiently than from 2D slices. Finally, we compared the performance of our model with and without cognitive estimation as a subtask. By adding cognitive estimation, the accuracy, *F*_1_-score and specificity were improved by 3.9, 4.4, and 8.5%, respectively, when GM was used as input. And similar improvements are seen when WM and GM were used as inputs, by 2.9, 3.7, and 4.8% on the accuracy, *F*_1_-score and specificity, respectively.

In terms of AUC, the cognitive estimation subtask brought a 4.8% improvement and also achieved the best classification performance (71.1%) of all models, further demonstrating its validity. This is consistent with the idea in previous studies that the association between brain abnormalities and cognitive symptoms may exist at a deep and abstract level and thus can be effectively captured by DL methods, leading to enhanced performance in EP classification (51, 52). These results demonstrate the effectiveness of using 3D-CNN and involving a cognitive estimation subtask for promising EP classification performance.

In addition, EP classification experiments were conducted on the external ABCD HCP-EP dataset to assess the robustness of the proposed method, and the results are presented in Table S4 in Supplementary material B. In general, the EP classification results of the proposed method on the external dataset are better than that on the in-house dataset, with an improvement of >4.6% on accuracy and >13% on *F*_1_-score when using WM and GM as inputs, and >3.8% on accuracy and >12.6% on *F*_1_-score when using only GM as input. Since the external dataset has more subjects than the in-house dataset, such an improvement suggests that even higher performance can be expected when more data is available. And more importantly, the proposed method also achieved higher EP classification accuracy and *F*_1_-score with the inclusion of the cognition estimation on the external dataset. Similar performance improvements from the inclusion of cognitive estimation were observed in both the in-house and external datasets, which again validates the effectiveness of the cognitive estimation subtask for facilitating EP classification.

### 4.3. Impact of cognition classification category quantity

The EP classification performance is largely unaffected in terms of F_1_ score and accuracy, while the specificity could be improved when *n* is set to ten. Therefore, in general, introducing a more challenging context in CLC subtask does not bring more discriminative information to the classification of EP. This may be due to the sample limitation in our study, when *n* is set to a large number, some categories may not have a sample at all. However, the improvement in specificity when *n* = 10 suggests that a larger number of categories may lead to better EP classification performance in the presence of abundant data.

### 4.4. Influence of WM/GM inputs

The model with GM as input outperformed the model with WM as input, with an improved F_1_ score of ≥5.6. This is consistent with previous results that EP causes significant changes in GM (48), while our results further indicate that changes in GM are sufficiently pronounced in EP and can significantly affect the performance of the automated classification tools. Even so, the simultaneous use of WM and GM achieves the best performance in most tasks, confirming the presence of both WM and GM alterations in patients with EP. Therefore, despite the best result was obtain when using only GM as input (i.e., 74.5% for EP + CLR), the inclusion of both GM and WM maps generally resulted in better classification performance for EP.

### 4.5. Influence of gender difference

Regarding the first experiment on gender difference that using gender information as input, the explicit inclusion has little effect on both cognitive estimation and EP classification tasks. For the second experiment that divided subjects into two subgroups, the overall performance decreased and the standard deviation increases sharply due to the small number of samples available for training the model in each subgroup. The model performance in the male subgroup are generally better than that in the female subgroup, as female subgroup has much fewer samples. Notably, in both subgroups, the EP classification performance still improved after the inclusion of cognitive estimation, suggesting that the effectiveness of including cognition estimation on facilitating EP classification was consistent, independent of gender differences.

### 4.6. Influence of CLC and CLR subtasks

Both CLC and CLR brought improvement on EP classification, while CLR seems to be more effective than CLC. The model incorporating the CLR subtask achieved the best performance on all metrics, with a significant gap compared to the other models. However, performance degrades when CLC is involved in addition to CLR, suggesting that the two subtasks may be incompatible. One possible reason for this is that some discriminative brain regions of the cognitive estimation dimensions may differ from the EP, thus introducing noisy features in the training. In contrast, the regression task did not bring discriminative information, so the features of CLR were more compatible than those of CLC in EP classification. In short, at least for EP classifications, the regression subtask is more informative than classification, but for other diseases the subtask needs to be selected on the merits (53, 54).

### 4.7. Interpretable sMRI biomarkers and clinical potentials

The entire GM structure contributes mostly to the EP classification, suggesting that more discriminative features are found in GM than in WM, which is in line with the results of better classification performance of the model using GM shown in the ablation study. Furthermore, for regions highlighted by GradCam of EP, saliency appears in the frontal and temporal lobe regions, as well as putamen, head of caudate nucleus, and thalamus. These regions contributed the most to our model in classifying a subject as an patient with EP or a healthy subject, suggesting that structural features in these regions are most likely to be discriminative for psychosis. Indeed, all of these regions recognized by our model are highly consistent with those reported in previous volumetric and functional studies. Alterations in gray matter, the frontal lobe, putamen, head of caudate nucleus, and thalamus were observed in patients with schizophrenia (55–58) and cognition deficits (59) in group-level volumetric analysis, as well as fMRI studies (60–62).

Interestingly, these regions are also consistent with those implicated in parvalbumin-expressing interneuron dysfunction (63–69), which is one core of schizophrenia pathophysiology, affecting neuronal synchronization and thalamocortical networks, and leading to cognitive deficits as well as hyperdopaminergia related to positive symptoms [reviewed herein (70)]. Taken together, our interpretable results indicate the potential of identifying biomarkers from sMRI by DL methods.

Moreover, some specific regions are also recognized as discriminative for the estimation of cognition level in the CLC subtask. Taking working memory as an example, the thalamus and cerebellum were highlighted by the DL model with the highest significance using GradCam, and these regions have also been proved to be associated with working memory function in the previous fMRI studies (71, 72). Similarly, in the result of ViL using GradCam, the highlighted regions of occipital lobe, thalamus, and cerebellum for visual learning were also considered associated to visual functions in fMRI studies (73–75). Furthermore, it is worth noting that the highlighted regions are not only different among the six different cognitive estimation dimensions, but differ significantly from those for EP classification. This could explain why CLC brings less improvement in EP classification than CLR, since the discriminative regions are different, the model may not be able to coordinate these features to accomplish both tasks simultaneously.

### 4.8. Limitation and future work

Although our proposed method achieves improved performance in EP classification and provides biomarkers with a high degree of interpretability, there are still some limitations that may affect the generalizability of our approach. First, the study was conducted at a single site and did not take into account the different ethnic composition and sMRI scanning settings, so multi-site studies are needed for further validation. Second, the EP subjects in our study received medication, which may also leads to structural alterations in the brain, thus requiring the use of a non-medicated sample in our future studies to rule out medication interference. Third, the study on the impact of gender differences may require a larger female group to further validate the proposed method.

Despite these limitations, our results also lead to many interesting directions for future research. For example, since only EP is studied in this work, whether the cognitive estimation subtask helpful for improving classification performance for other psychiatric disorders could be explored. And, as we demonstrated that implicitly introducing cognitive features in the DL model helps EP classification, the question is raised whether it is better to incorporate such additional features explicitly (i.e., as input) or implicitly (e.g., as output) into the workflow. Also, since deep learning and implicit information introduction can enhance classification, with only sMRI as a single input, more other relevant features can be introduced into the model in the same way with the aim of further improving classification performance and providing interpretable evidence to aid clinical translation. Moreover, if validated in larger cohorts of patients at the early phase of psychosis, this approach could open the way to prediction of cognitive deficit in prospective longitudinal study with patients in their prodromal phase.

## 5. Conclusion

In this study, we propose a multitask DL framework for EP classification based on sMRI images. By introducing cognitive estimation as a subtask, the proposed method is able to estimate the cognitive state of an individual and improve the classification performance of EP by an appreciable margin. Experimental results show that our method can not only achieve classification accuracy that exceeds that of the latest similar methods, but also identify discriminative regions in sMRI images as interpretable evidence.

## Data availability statement

The in-house dataset presented in this article are not readily available because the data that support the findings of this study are available from the corresponding author upon reasonable request. Requests to access the datasets should be directed to LX, lijing.xin@epfl.ch. For the publicly available ABCD HCP-EP dataset, please request access as per the official NIMH requirements.

## Ethics statement

The studies involving human participants were reviewed and approved by Commission Cantonale d'éthique de la Recherché sur l'être Humain-CER-VD. The patients/participants provided their written informed consent to participate in this study. The studies involving human participants were reviewed and approved by Cantonal Ethics Commission for Research on Human Beings. The patients/participants provided their written informed consent to participate in this study.

## Author contributions

YW: conceptualization, methodology, software, investigation, writing—original draft, and review. CZ: conceptualization, methodology, writing—review, editing, supervision, and funding acquisition. LC: supervision and funding acquisition. YD: methodology and investigation. MC and PC: data curation. RJ: data curation, writing—review, and editing. KD: data curation, writing—review, editing, resources, and funding acquisition. LX: conceptualization, writing—review, editing, supervision, resources, data curation, and funding acquisition. All authors contributed to the article and approved the submitted version.

## Footnotes

^1^ https://www.humanconnectome.org/study/human-connectome-project-for-early-psychosis

^2^ https://nda.nih.gov/ccf/

^3^ https://www.humanconnectome.org/storage/app/media/documentation/HCP-EP1.1/HCP-EP_Release_1.1_Manual.pdf

^4^pre-trained weights provided by torchvision package (version 0.7.0).

^5^ http://cobralab.ca/atlases/

^6^ https://scalablebrainatlas.incf.org/human/NMM1103

^7^ https://github.com/microsoft/LightGBM
