# Supplementary material for: Bridging structural MRI with cognitive function for individual level classification of early psychosis via deep learning
Source: Front Psychiatry. 2023 Jan 10;13:1075564. doi: 10.3389/fpsyt.2022.1075564 (PMC9871589; doi:10.3389/fpsyt.2022.1075564)
Supplement: Supplementary file 1 [file Data_Sheet_1.pdf]

Supplementary Material

A. PARTICIPANT RECRUITING CRITERIA

Inclusion criteria for EP were a) meeting the psychosis threshold as defined by the last subscale of the Comprehensive Assessment of At-Risk Mental States which allows to confirm that patients have crossed the psychosis threshold, b) no antipsychotic medication for >6 months, c) no psychosis related to intoxication or organic brain disease, and d) intelligence quotient >70. Healthy control participants must not a) meet criteria for a DSM Axis 1 and 2 disorder, b) be receiving any current treatment with psychotropic medication, c) have a family history of psychotic spectrum disorder.

B. RESULTS WITH HUBER LOSS

In addition to the MSE loss used for the CLR task, Huber loss Huber (1965) was also evaluated, which is defined as

$$\mathcal{L}_{Huber} = \begin{cases} \frac{1}{2}(g-\hat{g})^2 & if \ |(g-\hat{g})| < \delta \\ \delta((g-\hat{g}) - \frac{1}{2}\delta) & otherwise, \end{cases} \tag{S1}$$

where g, $\hat{g}$ and δ denote ground truth label, prediction, and interval, respectively. Compared to the MSE loss, the Huber loss is less sensitive to outliers because it only treats the error as square in the interval of δ, which is empirically set to 1.0 in our experiments. As shown in Table S2, Huber loss did not bring significant improvement compared to MSE loss, and in most cases even brought performance degradation. Furthermore, as shown in Table S5, the use of Huber loss in CLR also failed to bring a general improvement to the EP classification task. These results may be due to the fact that the cognitive data we used did not have many outliers to deal with, and a small interval δ may lead to gradient loss since some spurious outliers may be incorrectly excluded. Another possible reason is that since there is a new hyperparameter δ in the Huber loss, the empirically set value of 1 may not be the optimal value in all cases.

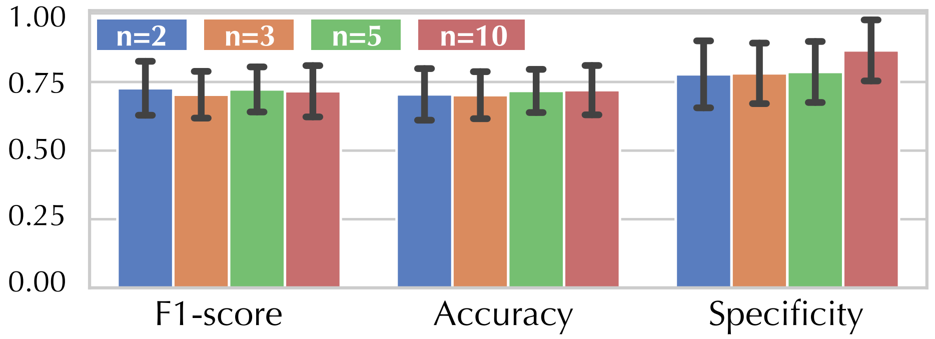

Figure S1. The EP classification performance of our model, when combined with different numbers of categories for CLC. Here n denotes number of categories.

Table S1. Results for cognition classification (F_1-score, %) and regression on average of six dimensions, here n denotes number of categories. The best results are in bold.

Models	CLC				CLR	
	n=2	n=3	n=5	n=10	R^2 ↑	MAE ↓
Volume + SVM	48.5 ± 11.3	31.0 ± 12.5	18.8 ± 8.6	8.0 ± 4.5	**-0.086 ± 0.139**	**7.299 ± 1.735**
Thickness + SVM	44.5 ± 9.7	29.0 ± 9.4	18.1 ± 7.2	8.3 ± 4.8	-0.108 ± 0.135	7.400 ± 1.725
Volume + DNN	66.2 ± 13.8	46.9 ± 10.6	31.2 ± 8.7	15.6 ± 4.0	-1.402 ± 0.932	10.360 ± 2.062
Thickness + DNN	66.6 ± 11.6	50.4 ± 7.8	**33.1 ± 6.9**	15.0 ± 3.8	-1.002 ± 0.821	9.588 ± 1.905
Image + RF	46.4 ± 11.6	32.2 ± 10.6	17.9 ± 8.7	8.9 ± 4.6	-0.202 ± 0.290	7.690 ± 1.481
Image + SVM	46.3 ± 11.6	30.2 ± 10.0	15.2 ± 7.4	7.2 ± 4.8	-0.123 ± 0.200	7.369 ± 1.732
Image + GBM	47.5 ± 11.5	35.0 ± 12.3	20.3 ± 9.7	8.5 ± 5.1	-0.667 ± 0.517	8.999 ± 1.971
Image + MNasNet (Tan et al., 2019)†	66.9 ± 3.4	50.6 ± 8.4	28.7 ± 8.8	15.2 ± 3.7	-0.881 ± 0.151	8.685 ± 1.945
Image + MNasNet (Tan et al., 2019)‡	67.8 ± 3.1	51.0 ± 7.4	29.0 ± 9.5	15.6 ± 3.8	-0.301 ± 0.302	8.601 ± 1.993
Image + ResNet-18 (He et al., 2016)†	63.7 ± 3.2	44.0 ± 7.9	25.7 ± 6.1	14.0 ± 3.0	-1.866 ± 0.978	17.318 ± 2.275
Image + ResNet-18 (He et al., 2016)‡	67.9 ± 4.9	51.1 ± 8.6	29.4 ± 7.3	15.1 ± 3.8	-0.493 ± 1.233	9.586 ± 1.439
Image + 3D-CNN (ours)	**70.1 ± 3.5**	**51.9 ± 8.1**	31.9 ± 7.5	**16.2 ± 3.7**	-0.878 ± 0.121	8.567 ± 1.950

†: train from scratch; ‡: using pre-trained weights.

Table S2. Results for CLR (in terms of R^2) on average of six dimensions using MSE and Huber loss. The performance gain or loss after using Huber loss compared to MSE loss is represented by ↑ and ↓.

Models	MSE loss	Huber loss
Image + MNasNet†	-0.881 ± 0.151	-0.694 ± 0.195 ↑
Image + MNasNet‡	-0.301 ± 0.302	-0.404 ± 0.212 ↓
Image + ResNet-18†	-1.866 ± 0.978	-1.922 ± 1.273 ↓
Image + ResNet-18‡	-0.493 ± 1.233	-0.331 ± 2.019 ↑
Image + 3D-CNN (ours)	-0.878 ± 0.121	-1.069 ± 0.434 ↓

Table S3. Results of F_1-score (%) for cognition classification and regression on average of six dimensions on ABCD HCP-EP dataset, here n denotes number of categories. The best results are in bold.

Models			F_1-score ↑		
	R^2 ↑	MAE ↓	n=2	n=3	n=5
Image + RF	0.043 ± 0.134	3.931 ± 0.784	52.6 ± 8.9	37.7 ± 9.9	19.0 ± 8.1
Image + SVM	-0.051 ± 0.197	4.418 ± 0.837	53.4 ± 8.8	42.1 ± 9.8	18.9 ± 7.6
Image + GBM	-0.129 ± 0.341	4.667 ± 0.809	52.4 ± 9.2	40.2 ± 9.9	19.4 ± 9.9
Image + MNasNet†	0.009 ± 0.084	3.294 ± 0.693	68.9 ± 3.8	55.6 ± 7.1	34.4 ± 8.1
Image + MNasNet‡	0.051 ± 0.109	**3.021 ± 0.623**	75.0 ± 3.1	60.3 ± 6.3	38.5 ± 7.9
Image + ResNet-18†	-0.093 ± 0.592	4.911 ± 0.599	72.4 ± 3.9	57.6 ± 6.3	35.2 ± 6.7
Image + ResNet-18‡	0.061 ± 0.403	4.137 ± 0.638	75.2 ± 3.2	60.2 ± 6.8	38.1 ± 6.4
Image + 3D-CNN (ours)	**0.074 ± 0.499**	3.867 ± 0.792	**81.6 ± 1.8**	**61.4 ± 5.9**	**40.3 ± 6.8**

†: train from scratch; ‡: using pre-trained weights.

C. COMPUTATIONAL COSTS

We further presented the cognition estimation performance of different models and compared their computational costs. As shown Table S6, the 2D-CNN model has at least two times more parameters compared to the 3D-CNN (ours), but at the same time the performance of cognitive estimation is lower. Instead of extracting features directly from the 3D sMRI volume, the 2D-CNN divided the volume into several slices for feature extraction and combined them into a very large feature

Table S4. Performance of EP classification on ABCD HCP-EP dataset. All results are shown in percentage and the best results are highlighted in bold.

Method	acc	F_1
sMRI images + 2D-CNN	75.3 ± 6.3	80.6 ± 4.9
Proposed w/o cognition assessment†	75.9 ± 5.3	84.1 ± 5.2
Proposed w/ cognition assessment†	78.1 ± 6.0	**87.2 ± 5.1**
Proposed w/o cognition assessment‡	75.8 ± 6.1	84.3 ± 5.1
Proposed w/ cognition assessment‡	**78.7 ± 5.9**	87.1 ± 5.0

†: WM and GM inputs; ‡: GM input.

Table S5. Results for EP classification incorporated with CLR. The performance gain or loss after using Huber loss compared to MSE loss is represented by ↑ and ↓.

Models	MSE loss		Huber loss	
	F_1	spe	F_1	spe
Image + MNasNet†	66.1 ± 5.1	70.3 ± 4.9	65.3 ± 3.1 ↓	69.9 ± 3.7 ↓
Image + MNasNet‡	68.0 ± 4.3	74.3 ± 5.3	67.7 ± 3.6 ↓	72.2 ± 4.4 ↓
Image + ResNet-18†	66.9 ± 3.4	72.3 ± 5.1	66.0 ± 4.2 ↓	73.2 ± 5.9 ↑
Image + ResNet-18‡	70.6 ± 6.1	77.9 ± 5.8	70.7 ± 4.8 ↑	77.2 ± 6.1 ↓
Image + 3D-CNN (ours)	74.5 ± 4.2	82.3 ± 6.3	74.3 ± 4.4 ↓	82.0 ± 6.2 ↓

Table S6. Results and model parameters for CLS (in terms of F_1) and CLR (in terms of MAE) tasks.

Models	F_1 ↑	MAE ↓	Parameters
Image + MNasNet	67.8 ± 3.1	8.6 ± 2.0	2.8M
Image + ResNet-18	67.9 ± 4.9	9.6 ± 1.4	11.3M
Image + 3D-CNN (ours)	70.1 ± 3.5	8.5 ± 2.0	1.2M
Image + 3D-CNN (1.5× neurons)	70.0 ± 4.9	8.8 ± 2.8	2.8M

embedding for nonlinear projection and final prediction, thus introducing more parameters than the 3D-CNN model. By learning features directly from the 3D volume, the 3D-CNN model not only has fewer parameters than the 2D-CNN model, but also provides better cognitive estimation performance. In addition, we made the parameters of 3D-CNN the same as those of MNasNet by increasing the number of neurons of 3D-CNN to 1.5 times of the original one. The modified 3D-CNN model achieved a F_1 score of 70.0 and MAE of 8.8, which is still better than all 2D-CNN models but worse than the original 3D-CNN model. This performance degradation may be due to an overfitting problem, since we have a relatively small amount of data.

D. DEEP LEARNING MODEL STRUCTURE

The deep learning model is designed to encode the input 3D sMRI scans into feature embeddings that are subsequently fed into several subbranches for cognitive estimation and EP classification. The encoding process is completed by five connected blocks, each of which consists of a convolutional layer, a batch normalization layer, a dropout operation (ratio 0.3), a leaky ReLU activation layer, and a max pooling layer. The encoded feature embeddings are then flattened (dimension 7168) and fed into the fully connected layer for nonlinear projection to predict category probabilities and continuous regression values. The stucture of the deep learning model can be found in Table S7.

Table S7. Details of the 3D-CNN architecture

Layer	Feature Channel	Stride	Kernel
Input	2		
Convolution	16	1×1×1	3×3×3
Batch Normalization	16		
Dropout (rate=0.3)	16		
Leaky ReLU	16		
Max Pooling	16	2×2×2	2×2×2
Convolution	32	1×1×1	3×3×3
Batch Normalization	32		
Dropout (rate=0.3)	32		
Leaky ReLU	32		
Max Pooling	32	2×2×2	2×2×2
Convolution	64	1×1×1	3×3×3
Batch Normalization	64		
Dropout (rate=0.3)	64		
Leaky ReLU	64		
Max Pooling	64	2×2×2	2×2×2
Convolution	128	1×1×1	3×3×3
Batch Normalization	128		
Dropout (rate=0.3)	128		
Leaky ReLU	128		
Max Pooling	128	2×2×2	2×2×2
Convolution	256	1×1×1	3×3×3
Batch Normalization	256		
Dropout (rate=0.3)	256		
Leaky ReLU	256		
Max Pooling	256	2×2×2	2×2×2
Fully Connected	7168		
Dropout (rate=0.3)	7168		
Softmax	2		
Output (Schizophrenia)	2		
Fully Connected	7168		
Dropout (rate=0.3)	7168		
Softmax	6		
Output (Cognition Classification)	6		
Fully Connected	7168		
Dropout (rate=0.3)	7168		
Output (Cognition Regression)	6		

Table S8. Performance comparison on cognition estimation and EP classification using sMRI scan and gender embedding as inputs.

Method	Cognition Estimation		EP Classification	
	R^2 ↑	F_1-score (n=2) ↑	acc ↑	F_1-score ↑
Image + MNasNet†	-0.301 ± 0.302	67.8 ± 3.1	-	-
Image + MNasNet‡	-0.299 ± 0.294	67.7 ± 3.1	-	-
Image + ResNet-18†	-0.493 ± 1.233	67.9 ± 4.9	-	-
Image + ResNet-18‡	-0.489 ± 1.241	68.0 ± 4.7	-	-
Image + 3D-CNN†	-0.878 ± 0.121	70.1 ± 3.5	-	-
Image + 3D-CNN‡	-0.885 ± 0.126	70.0 ± 3.5	-	-
Proposed w/o cognition assessment†	-	-	71.0 ± 4.3	70.1 ± 4.4
Proposed w/o cognition assessment‡	-	-	70.9 ± 4.4	70.0 ± 4.4
Proposed w/ cognition assessment†	-	-	74.9 ± 4.3	74.5 ± 4.2
Proposed w/ cognition assessment‡	-	-	74.8 ± 4.3	74.5 ± 4.3

†: without gender embedding; ‡: with gender embedding.

Table S9. Performance comparison of EP classification for different gender subgroups.

Method	Male (62 subjects)		Female (15 subjects)	
	acc	F_1-score	acc	F_1-score
Proposed w/o cognition assessment†	70.0 ± 6.1	70.1 ± 5.8	66.3 ± 10.3	65.9 ± 9.7
Proposed w/ cognition assessment†	73.0 ± 5.8	74.3 ± 5.1	68.1 ± 9.8	68.4 ± 9.6
Proposed w/o cognition assessment‡	70.8 ± 6.4	70.0 ± 5.6	66.4 ± 9.9	66.1 ± 10.4
Proposed w/ cognition assessment‡	74.6 ± 6.7	74.1 ± 6.0	68.7 ± 10.2	69.0 ± 8.9

†: WM and GM inputs; ‡: GM input.

REFERENCES

He, K., Zhang, X., Ren, S., and Sun, J. (2016). Deep residual learning for image recognition. In *Proceedings of the IEEE conference on computer vision and pattern recognition.* 770–778

Huber, P. J. (1965). A robust version of the probability ratio test. *The Annals of Mathematical Statistics* , 1753–1758

Tan, M., Chen, B., Pang, R., Vasudevan, V., Sandler, M., Howard, A., et al. (2019). Mnasnet: Platform-aware neural architecture search for mobile. In *Proceedings of the IEEE/CVF Conference on Computer Vision and Pattern Recognition.* 2820–2828
